# Building capacity for maternal, newborn and child health research in low-income country settings: A research fellowship experience in Ethiopia

**DOI:** 10.7189/jogh.14.04198

**Published:** 2024-11-29

**Authors:** Kassahun Alemu, Lisanu Taddesse, Clara Pons-Duran, Bezawit Mesfin Hunegnaw, Robera Olana Fite, Abebe Belayneh Bekele, Frederick GB Goddard, Assaye K Nigussie, Yifru Berhan, Delayehu Bekele, Theodros Getachew, Ebba Abate, Getachew Tollera, Grace J Chan

**Affiliations:** 1 HaSET Maternal and Child Health Research Program, Addis Ababa, Ethiopia; 2 Department of Epidemiology, Harvard T.H. Chan School of Public Health, Boston, Massachusetts, USA; 3 Department of Pediatric and Child Health, Saint Paul's Hospital Millennium Medical College, Addis Ababa, Ethiopia; 4 College of Medicine and Health Sciences, Bahir Dar University, Bahir Dar, Ethiopia; 5 Department of Obstetrics and Gynecology, Saint Paul's Hospital Millennium Medical College, Addis Ababa, Ethiopia; 6 Health System and Reproductive Health Research Directorate, Ethiopian Public Health Institute, Addis Ababa, Ethiopia, Addis Ababa, Ethiopia; 7 Director General, Ethiopian Public Health Institute, Addis Ababa, Ethiopia; 8 Deputy Director General Office for Research and Technology Transfer Directorate, Ethiopian Public Health Institute, Addis Ababa, Ethiopia; 9 Department of Pediatrics, Boston Children’s Hospital, Harvard Medical School, Boston, Massachusetts, USA

## Abstract

**Background:**

There is a need to build research capacity to improve maternal, newborn, and child health (MNCH) in low- and middle-income countries (LMICs). To address this gap, we co-designed the HaSET (meaning ‘happiness’ in Amharic) MNCH Research Fellowship programme for academics and policymakers in collaboration with the Ministry of Health (MOH) and academic institutions in Ethiopia.

**Methods:**

Based on interviews and focus group discussions regarding a landscape analysis of the MNCH research environment, we developed an innovative ‘learning by doing’ model in which fellows identified research questions, developed proposals, obtained institutional review board (IRB) approvals, conducted research, analysed data, disseminated their findings, and developed policy briefs. Postdoctoral fellows were paired with policymakers and health professionals at the MoH to facilitate the translation of research findings into policy and programmes. Each pair received mentorship from a member of the HaSET’s scientific advisory group (SAG) who had expertise in research methods, data analysis, dissemination, and translation of evidence into policy.

**Results:**

The HaSET MNCH Research Fellowship curriculum included 10 modules covering topics from biostatistics to study operations and professional development. From March 2021 to July 2023, five postdoctoral fellows from local universities and four policymakers from the MoH and government research institutes underwent the HaSET programme, where they learned to gather high-quality evidence on priority research questions and guide the implementation of national policies and programmes. Leveraging existing data, the fellows produced 15 manuscripts and 11 policy briefs. The programme established a functional research link between the MoH, regional health bureaus, and local universities, while utilising the SAG’s expertise in mentorship.

**Conclusions:**

This robust and comprehensive HaSET MNCH Research Fellowship produced the first cohort of dedicated fellows trained in evidence-based medicine and mentored them to become effective public health professionals. They conducted high-quality studies to inform policy decisions on MNCH interventions in Ethiopia. Given its sustainability and scalability, researchers and academic institutions can further adapt the fellowship curriculum within capacity-building programmes to educate the next generation of research leaders in LMICs.

Strategies to achieve the Sustainable Development Goals (SDGs) aimed at reducing maternal and child mortality include promoting research and capacity-building initiatives to increase the quality and use of scientific evidence [1]. In this sense, locally-driven and locally-generated health research is crucial for addressing global health challenges [2–4]. In low- and middle-income country settings (LMICs), however, limited research capacity prevents individuals, organisations, and societies from gaining, enhancing, and sustaining the skills and resources needed to set and achieve their development goals over time [1,5–7]. Several existing initiatives have focussed on building research capacity to develop a strong cadre of researchers in LMICs [2,3,8–11], with the successful ones typically involving collaboration and networking, bringing in more policymakers in the capacity-building process, fostering newly-identified research competencies; and being highly focussed on specific research priorities [12–14].

Challenges in building research capacity stem from a lack of knowledge or skill in research problem identification, advanced study design, techniques, and methods, data analysis, dissemination, and the application of research findings [15–18]. Moreover, low-resource settings usually do not place priority on research, which is why they often have poor research infrastructure, such as limited access to academic resources, inadequate mentorship and training, insufficient academic networks, inability to obtain up-to-date research and knowledge, and weak or severely limited career progression pathways for researchers [9,19–21].

To address identified gaps and barriers in building research capacity in Ethiopia, the Ethiopian Public Health Institute (EPHI), St. Paul's Hospital Millennium Medical College (SPHMMC), Ministry of Health (MOH), Regional Health Bureaus, and the Harvard T.H. Chan School of Public Health collaboratively established a fellowship programme as part of the HaSET (meaning ‘happiness’ in Amharic) Maternal and Child Health Research Program. The main objective was to train a cohort of researchers in maternal, newborn, and child health (MNCH) who would, through the training programme, generate high-quality evidence to inform decision-making and national policy and programme implementation.

The HaSET MNCH Research Fellowship aimed to promote a culture of evidence-based decision-making throughout the research life cycle, from problem identification, study analysis, and implementation to policy formulation and programme development. The fellowship focussed on generating relevant high-quality evidence, robust analysis, and the use of data in supporting policymaking efforts to improve maternal and child health and well-being.

## METHODS

### Assessment of MNCH research capacity and needs

We conducted a landscape analysis of the MNCH research capacity in Ethiopia across each component of research: formulation of research questions, study design, study implementation, data analysis, dissemination, and translation to policy. To achieve this, we held key informant interviews and focus group discussions with a variety of government and academic stakeholders with experience on MNCH issues [22,23]. The aim was to assess whether the current research capacity landscape in the country is strong or limited. This was followed by a consensus building workshop that included stakeholders to agree on key MNCH research objectives. Details on these processes are available in a previously published scoping review [22] and a research environment landscape analysis (manuscript not yet published).

### Curriculum and implementation modalities

We developed the core curriculum by identifying the following key components of MNCH research: formulation of research questions, study design, study implementation, data analysis, dissemination, and translation to policy and public health programming. We designed a module and a full syllabus for each component, including prerequisites, specific learning objectives, methods for achieving those objectives, instructional resources, and deliverables. Each module was led by a team of international and local experts who were part of the HaSET MNCH Research Fellowship team to ensure the integration of learning objectives and consistency between modules.

### Scientific advisory group

We then established a scientific advisory group (SAG) to mentor the HaSET fellows. The SAG comprised senior-level academics and researchers with a strong foundation in MNCH fields, including obstetricians and gynaecologists, paediatricians, child health specialists, epidemiologists, and public health experts in MNCH. HaSET’s SAG included experts representing Addis Ababa University, Jimma University, EPHI’s National Data Management Center, SPHMMC, and Harvard Chan School of Public Health. We selected the members based on their extensive knowledge and experience in health research in Ethiopia and their commitment to helping the HaSET fellows develop as researchers. They then had to consent to our Terms of Reference (TOR), which provided a framework outlining their advisory roles as mentors to HaSET fellows and other expected interactions and contributions to the HaSET programme. Quarterly review meetings were held with the SAG during the term of the investment, including virtual meetings during the coronavirus disease 2019 (COVID-19) pandemic and subsequent in-person meetings.

Each SAG member agreed to dedicate two hours per week to support the development of the HaSET deliverables, including manuscripts and policy briefs. SAG meetings followed a consistent format and typically included a review of HaSET programme progress, updates from the Birhan field site, and reports on the performance and progress of the HaSET MNCH Research Fellowship where HaSET fellows presented their MNCH research outputs, including the research proposals, protocols, analysis, manuscripts, and policy briefs.

HaSET also established a steering committee (SC) with representatives from EPHI, MOH, SPHMMC, SAG, the Birhan Community Advisory Board, and Harvard University. The SC convened annually during the term of the investment according to its own TOR. Both the HaSET’s SAG and the SC were instrumental in fostering a collaborative research approach, where researchers from academic, governmental, and community settings were linked to increase local research capacity, establish an MNCH research hub, and conduct high-quality, high-impact MNCH research.

### Postdoctoral and implementation fellows

The HaSET MNCH research fellows were recruited from two distinct groups: postdoctoral research fellows and policymakers (referred to as implementation fellows in the manuscript). The postdoctoral fellows mainly came from among early career faculty members at Ethiopian universities, while the implementation fellows were mostly early/mid-level health professionals engaged in the health care system at policy-developing or implementing institutions, such as the MoH, Regional Health Bureaus, and the EPHI (**Table 1**).

**Table 1:** Postdoctoral and implementation fellows’ recruitments criteria for the HaSET MNCH Research Fellowship, Ethiopia, November 2020 to January 2021

Postdoctoral fellows	Implementation fellows
PhD qualification in epidemiology, statistics, public health, or medical doctor with specialty and/or interest in MNCH research.	Early/mid-level health professionals holding an MSc/MPH degree and a minimum of two years’ experience with MNCH programme management.
Experience in research implementation, research design, sampling approaches and sample size estimation, data collection, data analysis, and field work.	Experience in policy implementation, writing policy briefs and translating evidence to policy guidance related to MNCH and writing policy implementation plans.
Excellent analytical, organisational, and problem-solving skills.	Field experience of data collection, data management, analysis, and organisational/problem-solving skills.
Written commitment from the host institution for their full-time availability during the two-year HaSET MNCH research fellowship, with an agreement to return to the host institution after the fellowship programme.	Written commitment from the host institution for the availability of 20% of their time during the two-year HaSET MNCH research fellowship, with an agreement to return to the host institution after the fellowship programme.

MNCH – maternal, newborn, and child health

At the initial stage of the three-step recruitment process, the applicants were assessed for eligibility based on screening of required documents such as academic credentials, cover letter, curriculum vitae, concept notes, and recommendation letters. At the subsequent stage, they were for their credentials, curriculum vitae, and research statements. A select group of applicants were invited to the final stage, which involved interviews and presentations of research questions and proposed approaches. The SAG and HaSET MNCH Research Fellowship team independently scored and discussed the applicants, and then reached a consensus to select the postdoctoral and implementation fellows.

### Fellowship deliverables

To achieve the project's deliverables, all fellows first identified a high-priority research question of significance to the MoH. They were responsible for developing and receiving approval for concept notes, proposals, and protocols for all research activities, as well as ensuring technical and ethical review and amendments from the institutional review boards (IRBs) of the partner academic institutions. They were also required to develop and receive approval for the analysis plan, as well as data analysis codes which were subsequently quality-checked. Writing retreat workshops were held for development of the deliverables where the fellows received support from SAG mentors, coauthors, and the HaSET MNCH Research Fellowship team. A writing coach provided critical reviews throughout the process of developing the manuscripts.

## RESULTS

### MNCH research capacity and needs

**Figure 1:**
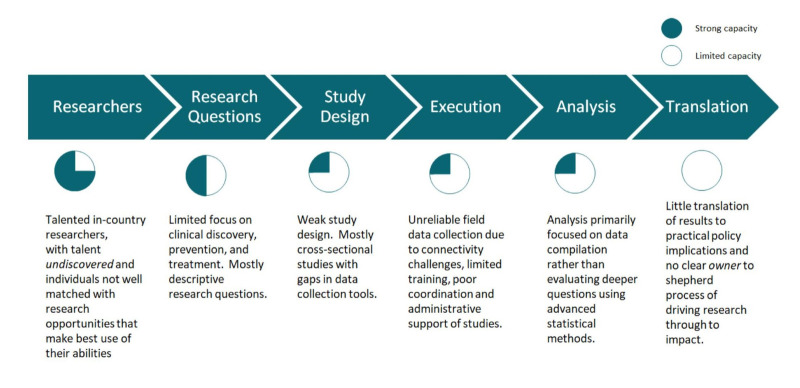
Maternal, newborn, and child health research landscape in assessment, HaSET MNCH Research Fellowship, Ethiopia, 2019.

Based on key informant interviews and focus groups, we identified that the researchers had limited capacity to formulate research questions on clinical discovery, prevention, and treatment, as well as difficulty in choosing and implementing appropriate study designs, apart from cross-sectional studies. There was poor coordination and administrative support of studies and gaps in designing data collection tools and in collecting data, including limited training. Moreover, the researchers had limited skills in formulating more complex research questions, using advanced statistical methods, and in translating results to practical policy. There was no clear owner or leading organisation to guide the process of driving research through to impact (**Figure 1**).

### Curriculum and implementation modalities

The fellowship objectives focussed on identifying research questions to address gaps, developing a network of MNCH researchers, conducting research studies, and building capacity through an innovative application of the ‘learning by doing’ model, where trainees gain knowledge and skills through hands-on, relevant practical experience, rather than through an passive, theory-focussed learning approach. Key components of the ‘learning by doing’ model were regular meetings for experience sharing and lessons learned among the SAG and the HaSET fellowship team, dedicated and continuous mentorship, and workshops at each phase of the research study.

The HaSET MNCH Research Fellowship curriculum included 10 modules covering orientation, the translation of research into policy, research methodology, ethics, epidemiology, biostatistics and data analysis, study operations, data collection and management, research communication, and professional development (**Table 2**).

**Table 2:** Summary of HaSET MNCH Research Fellowship curriculum with core learning objectives, deliverables, and methodology, March 2021 to July 2023

Modules	Learning objectives	Deliverables	Methodology
Orientation	Understand the fellowship objectives, governance structure, and coordination mechanisms; learn about the fellowship program objectives and implementation arrangements; understand the state of MNCH research in Ethiopia; understand initiatives related to research translation to policy and research in collaboration between government, research, and academic institutions.	List major health system and service delivery challenges that can be addressed through implementation research and clinical trials; identify vulnerable populations for essential MNCH services in Ethiopia, the demand side barriers to healthcare, and the pathway to care.	Overview; focussed lecture; group work; independent work; review; module lead feedback for each fellow
Translating evidence to policy	Develop a concept note on evidence-based operational and strategic plan; conduct a policy gap analysis to design a research question and objectives; design and conduct quality research using a standard research methodology and quality assurance tool; develop a detailed process and implementation plan of phases of an operational feasibility study; develop a concrete action plan for research dissemination, advocacy, and communication for research translation to action.	Identify lists of priority MNCH policy and implementation gaps in Ethiopia; develop an original research question and concept note based on policy gaps; develop a model for an operational feasibility study plan; develop an outline for a policy brief note.	Overview; focussed lecture; group work; independent work; review; module lead feedback for each fellow.
Research methodology	Develop a research protocol; conduct a narrative scoping review (a non-systematic comprehensive and objective analysis of the current knowledge on a topic); frame an effective research question; develop concept notes describing rationale and methodology; conduct a systematic review and meta-analysis; identify a review question(s); design a systematic search strategy; systematically search and review published and unpublished literature from databases and search engines; systematically analyse published data using either qualitative tools or quantitative meta-analyses.	Identify research question(s) for systematic review; develop a systematic review protocol according to PRISMA-P checklist; complete a literature review focussed on the research question; complete a systematic review and meta-analysis according to PRISMA checklist.	Overview; focussed lecture; group work; independent work; review; module lead feedback for each fellow.
Ethics	Understand ethical responsibility of researchers to study participants; scientific merit of the research, ethical justification, and acceptance; privacy; conducting research in biologically vulnerable population, including pregnant women, young children, prisoners, etc; IRBs approval.	Group case discussion from World Health Organization’s Casebook on Ethical Issues in International Health Research; develop a summary of the case discussion; complete an individual consent form relating to a fictional research proposal.	Overview; focused lecture; group work; independent work; review; module lead feedback for each fellow.
Epidemiology	Compute and interpret epidemiologic measures of disease frequency; select and apply appropriate measures of association and measures of public health impact; design, analyse and evaluate epidemiological study designs; identify sources of biases (selection, information, and confounder) at all stages and design strategies to minimise at design and analysis stages; quantify the effects of biases and uncontrolled confounding; stratify analyses to assess effect modification and interaction; design and evaluate maternal and perinatal death surveillance and response; understand MNCH epidemiologic profile, gaps, and inform study designs.	Select appropriate measures of disease frequency and association for specific epidemiologic study topics; provide critical appraisals of study design; direct acyclic graphs for the selected study proposals; provide critical appraisal of bias in observational studies; participate in group exercise on public health surveillance with emphasis on maternal and perinatal death surveillance and response; conduct and apply mediation analysis, identify mediators, and interpret in fellows’ research; complete individual exercise on validity in epidemiologic studies – critical appraisal of selected materials; complete critical appraisal of published articles, policies, strategies on implementation epidemiology.	Overview; focussed lecture; group work; independent work; review; module lead feedback for each fellow.
Biostatistics and data analysis	Understand a wide range of statistical methods to explore and analyse their data and perform analyses in R: summarise data using numerical and graphical summaries; conduct an exploratory data analysis to better understand the nature of their data; utilise their understanding of basic probability theory, random variables, and common distributions; carry out inference for categorical and numerical data; carry out inference in the regression context, including multiple linear regressions and multiple logistic regressions; conduct survival analysis; carry out inference in the context of cluster-correlated/longitudinal data; analyse complex survey data; develop an analysis plan for their research project(s).	Complete analysis plan; participate in a peer review of analysis plans; conduct preliminary data analysis; conduct baseline data analysis; conduct end line data analysis.	Overview; focussed lecture; group work; independent work; review; module lead feedback for each fellow.
Study operations	Understand study protocol and standard operating procedure; define basic concepts of designing a protocol; describe the purpose and main elements of a research protocol; develop a scientifically rigorous protocol that answers the research questions with transparency of methodologies and study designs; develop a standard operating procedure: step-by-step instructions to carry out research as per protocol; achieve quality output; reduce miscommunication; and increase uniformity of the performance; understand study management; develop a trainer’s guide and conduct a training workshop to train the research team (data collectors, supervisors, data management, laboratory staff and others) including the data collection pilot test to ensure data quality; develop a work plan and a monitoring and supervision plan to ensure that study objectives can be delivered on time and with high quality; develop tools to support study implementation, such as a site readiness checklist and supervision checklists; develop and apply budget guidance and financial and procurement guidelines; assess the resource management practice in relation to efficiency and effectiveness; draft a study budget, procurement plan, and budget monitoring tools to ensure proper cost controls; understand different types of study reporting and their audiences (e.g. results, funder reporting, financial reporting).	Develop a protocol; develop the step-by-step standard operating procedures; develop a work plan and monitoring and supervision plan for their research; develop a budget and financial and procurement management guideline.	Overview; focussed lecture; group work; independent work; review; module lead feedback for each fellow.
Data collection	Define and align the type of data sources, data collection methods and procedures with the outcome and exposure variables of the research objectives; select and develop instruments/tools/scales for measuring the intended variables/ constructs; compute index of content validity and translate tool/instrument/scale into local language (forward and backward translation) with expert panel discussions; design and apply electronic data collection conduct full psychometric test for the purposes of criterion, construct, convergent and concurrent validity checks; test the reliability of data collection tools (internal consistency, test-retest reliability, and inter-rater reliability): plan and implement field data collection activities including recruitment and training of study staff, adherence to protocol, supervision and mentorship.	Choose and define data sources, collection methods and procedures that fit with the fellow’s research proposal; selecting and developing the scales/tools/instruments to answer the fellow’s research questions; convert survey instrument into electronic form for mobile data collection; validate and test the reliability of the tools by conducting cognitive evaluations; develop a data collection SOP.	Overview; focussed lecture; group work; independent work; review; module lead feedback for each fellow.
Data management	Understand and implement the lifecycle of data management processes from planning and data collection through dissemination and preservation of research data; data acquisition and capture; data backup and recovery; data management and maintenance; data retention or destruction; design strategies/protocol on data quality assurance on how to maintain it by identifying inconsistencies and other anomalies in the data, and performing data cleaning activities to improve the data quality.	Fellow’s outline and present data management process/plan for their own research study; fellows will develop a data quality SOP and prepare standardized training manuals to document measurement protocols, detail procedures, and minimize errors.	Overview; focussed lecture; group work; independent work; review; module lead feedback for each fellow.
Research communication	Written communication; outline key ideas according to the specific content of a range of professional papers identified as HaSET programme deliverables; grant proposals; scientific papers using quantitative and qualitative methodology; systematic reviews and meta-analysis; abstracts (for conference submission and manuscripts); magazines, newspapers, and online media articles; construct and combine sentences and paragraphs into concise and clear papers identified as HaSET programme deliverables; revise draft papers based on edits and comments from peers and supervisors until ready for publication and dissemination; write a summary of a specific paper to submit to a conference, symposium event, or professional meeting to be chosen as either an oral presentation, poster presentation or an oral poster presentation; develop a poster based on the conference, symposium event, or professional meeting’s particular instructions; understand ethical issues associated with science research and with scientific publishing, including open access and data sharing; understand the editorial processes, including the external peer-review system and how to respond to edits/comments and developing a clean version of the paper.	Develop a competitive grant research proposal according to the specified criteria; develop a systematic review/meta-analysis paper according to the specified criteria; develop a policy brief focused on evidenced-based findings and provide relevant recommendations for policy makers; develop a scientific manuscript ready for submission to a peer reviewed national/international journal; prepare and deliver a presentation for a conference, symposium event, or professional meeting.	Overview; focussed lecture; group work; independent work; review; module lead feedback for each fellow.
Oral communication	Develop and present an oral presentation based on a research paper; develop and present an oral poster presentation.		Overview; focussed lecture; group work; independent work; review; module lead feedback for each fellow.
Professional development	Understand the fundamental principles and core competencies of professional development; identify different approaches for continuous learning and capacity building for professional development; conduct self-assessment on professional development and identify essential skill and competency gaps; identify the personal USP; design and develop a career and professional development strategy; develop a tool to track progress on professional development.	Conduct assessment on career and professional development; report on findings to group; develop a personal career and professional development strategy; produce a career and professional development monitoring tool.	Overview; focussed lecture; group work; independent work; review; module lead feedback for each fellow.

IRB – institutional review board, MNCH – maternal, newborn, and child health, SOP – standard operating procedures, USP – unique selling points

The fellowship curriculum was available online through the Canvas learning management system to facilitate virtual access anytime and anywhere. All lectures, reading materials, slide decks, and resources were made available through Canvas and Google Drive. Modules and workshops, meanwhile, were delivered using a hybrid approach (i.e. both virtually and in person), with most being delivered physically in Addis Ababa, Ethiopia. Each module used different teaching strategies, which were described in depth in its individual syllabus and directly by the module leads. The main learning methodologies were lectures, small and large group work with problem-solving and critical thinking activities, and review and mentoring time. As part of the research communication and professional development modules, the fellows wrote their research papers and presented and defended their research findings.

Each fellow had a time plan agreed upon with their SAG mentor. They were evaluated on a weekly, monthly, and quarterly basis to track their progress. Additional online training sessions, workshops, and retreats were conducted to strengthen technical skills, such as code management, longitudinal data management and analysis, and human subjects’ research.

### Fellows

Forty-five candidates applied for the postdoctoral fellows' positions; following the screening process, 12 individuals were shortlisted for further consideration. Five postdoctoral fellows who met all the criteria were subsequently recruited and enrolled.

Thirty-nine candidates applied for the implementation position, including 25 from the MoH, three from the Amhara Regional Health Bureau, and 11 from the EPHI. Five implementation fellows were recruited: three from the MoH, one from the EPHI, and one from the Amhara Regional Health Bureau.

Of the ten selected postdoctoral and implementation fellows, five were women, including one postdoctoral and four implementation fellows. From March 2021 to July 2023, these individuals underwent training within the HaSET MNCH Research Fellowship to enhance MNCH research in their country.

### Fellowship deliverables

The following success metrics were established for the HaSET project: a systematic review/meta-analysis, a manuscript conducting a secondary analysis of existing data, and a policy brief authored by the fellowship pair. The postdoctoral and implementation fellow pairs developed five SRMA protocols, all of which were registered in PROSPERO. Three systematic reviews and three meta-analyses manuscripts (six in total) were completed.

The fellows developed nine analysis plans for the manuscripts based on secondary analyses of existing data, which were reviewed and approved for statistical soundness. They also completed a data use agreement for acquiring data from the Birhan Health and Demographic Surveillance System cohort [23] and produced approved codes for both these manuscripts and their systematic review/meta-analysis, which were used as quality checks after they reviewed the populated results in texts, graphs, and tables to ensure their appropriateness.

Eleven policy briefs were produced from the results of studies that primarily focussed on synthesising Ethiopian-based empirical evidence. The 15 manuscripts (nine from the secondary analyses and six from systematic review and meta-analyses) were submitted to a special thematic research collection of the Journal of Global Health and are currently at various stages of the review process. Nine fellows graduated from the fellowship programme after completing the curriculum modules, the manuscripts, and policy deliverables. One implementation fellow transferred to a non-governmental organisation during the fellowship and did not complete the fellowship.

## DISCUSSION

Using an innovative approach, the HaSET MNCH Research Fellowship programme combined academic rigor with policy impact, thereby becoming the first postdoctoral research programme in maternal and child health in the history of Ethiopia. To narrow the gaps that had been identified in the initial landscape assessment of research capacity and needs, the fellowship built local research capacity by focussing on identifying and conducting MNCH-priority research. Fellows were recruited from academia and the national health system, including the MoH, Amhara Regional Health Bureau, and the EPHI. The innovative ‘learning by doing’ curriculum was guided by principles of data-driven, user-centred, integrated, and outcome-focussed research, with all modules being developed and delivered by international and local experts in the field.

In parallel with the curriculum, postdoctoral fellows partnered with implementation fellows from the MoH, EPHI, and regional health bureaus to undertake high-priority research studies. Based on a ‘learning by doing’ approach driven by peer learning, mentoring, and coaching, the fellows received support for study design and data analysis while focussing their work on national health priorities. The product were high-quality results which soon led to significant policy impact [15]. The SAG provided regular quality reviews periodically, demonstrating how a tailored approach to competency-based education could improve and address various phases of the competency continuum [24,25]. Employing this mentoring approach allowed fellows to be linked and networked into the existing government, research, and academic institutes.

This initiative was based on cross-country collaborations and networking and involved stakeholders in the capacity building process, including identifying competence and setting highly focused research priorities [12–14]. The guidance and leadership provided by the MoH were crucial elements in the success of the HaSET MNCH Research Fellowship. The MoH and the MNCH leading executive offices under MoH have been invaluable advisors from the outset, volunteering their involvement, guidance, and support in setting priorities and selecting and ensuring that fellows had sufficient leave from their demanding work schedule to actively participate in research. Additionally, the SAG’s contributions in providing mentorship and coaching to the fellows throughout the fellowship have been vital. However, the effects might be limited, which are not supported by primary data from fellows, SAGs, and hosting institutions.

## CONCLUSIONS

The HaSET MNCH Research Fellowship successfully trained the first cohort of postdoctoral and implementation fellows in Ethiopia based on evidence-based medicine, to be monitored and further mentored in the future. This fellowship programme provided a conducive environment for faculty members and public health leaders to mentor fellows, resulting in the development of competent public health professionals. It also established a strong research link between the government, academia, and public health experts through the mentoring approach. Based on these findings, we believe there is potential to scale the fellowship and implement it elsewhere to sustain successful capacity building programmes, thereby building the next generation of leaders in research in LMICs.
